# Funk & Wagnalls’ Standard Dictionary of the English Language

**Published:** 1894-01

**Authors:** 


### FUNK & WAGNALLS' STANDARD DICTIONARY OF THE ENGLISH LANGUAGE. Volume I of this great work will be issued on December 16th.

This volume has been four years in making; two hundred and thirty-eight editors and specialists have been employed upon it, and the cash outlay has been about a half million dollars. The advance orders for the work mount up into the tens of thousands.

The following letter was received by the publishers from a well-known gentleman, prominently identified with the late World's Fair at Chicago:

MINES AND MINING BUILDING,
JACKSON PARK, ILL.

*Messrs. Funk & Wagnalls.*

GENTLEMEN:—I am pleased to inform you that the *Standard Dictionary* has been granted an award (diploma and medal) in group No. 150. The exact wording of all the awards will not be announced for probably three or four weeks.

The Dictionary exhibit consisted of a number of proof-sheets, as the work was only part in type. This fact makes the award more significant. The award of diploma and medal is the only class of awards granted. A gentleman who was present during the examination informs the publishers that the judges devoted nearly three hours to a critical inspection of the sheets (it was a very unusual thing to devote so much time to the examination of any exhibit), comparing the definitions with those of other dictionaries, and that they frequently expressed themselves as highly pleased and in favor of the features of the *Standard*. At the close of the examination one of the judges remarked: "I have the best of other dictionaries, but this work has desirable features that others have not. I must possess a copy when it is published."

The vocabulary of the *Standard* is extraordinarily rich and full, that of no other dictionary nearly equalling it, although great care was taken to throw out all useless words.

The following is an actual count of words and phrases recorded under the letter A:

| | |
|---|---|
| *Stormonth Dictionary*, total terms in A, . . . . . . . . . . . . . . . . . . . | 4,692 |
| *Worcester Dictionary*, total terms in A, . . . . . . . . . . . . . . . . . . . | 6,983 |
| *Webster* (*International*) *Dictionary*, total terms in A, . . . . . . . . . . . . . | 8,358 |
| *Century Dictionary*, total terms in A, . . . . . . . . . . . . . . . . . . . . . | 15,621 |
| *The Standard Dictionary*, total terms in A, . . . . . . . . . . . . . . . . . | 19,736 |

The full number of words and terms in these dictionaries for the entire alphabet is as follows: *Stormonth*, 50,000; *Worcester*, 105,000; *Webster* (*International*), 125,000; *Century* (six volumes, complete), 225,000; *Standard*, 300,000.

